# NDRG4 stratifies the prognostic value of body mass index in colorectal cancer

**DOI:** 10.18632/oncotarget.6182

**Published:** 2015-10-20

**Authors:** Jianyong Zheng, Yunming Li, Shaojun Zhu, Jipeng Li, Qingchuan Zhao, Gang Ji, Weizhong Wang, Dake Chu

**Affiliations:** ^1^ State Key Laboratory of Cancer Biology and Xijing Hospital of Digestive Diseases, Xijing Hospital, Fourth Military Medical University, Xi'an, China; ^2^ Department of Health Statistics, Fourth Military Medical University. Xi'an, China; ^3^ Department of Pathology, Fourth Military Medical University. Xi'an, China; ^4^ Department of Biochemistry and Molecular Biology, State Key Laboratory of Cancer Biology, Fourth Military Medical University, Xi'an, China

**Keywords:** NDRG4, colorectal cancer, obesity, disease-free survival, overall survival

## Abstract

NDRG4 is a novel candidate tumor suppressor and can inhibit PI3K/AKT signal which is related with energy balance and related carcinogenesis. In the present study, we investigated whether NDRG4 status could modify the association of obesity with clinical outcome of colorectal cancer. For this purpose, a hospital-based prospective study cohort of 226 colorectal cancer patients was involved. NDRG4 mRNA levels were determined by real-time PCR. Association of NDRG4 mRNA expression with disease-free and overall survival was studied first. Then, the association of obesity with clinical outcome was determined according to NDRG4 level. Multivariate Cox proportional hazards model was used to compute hazard ratio, adjusting for covariates including microsatellite instability, KRAS, BRAF and PIK3CA mutation. Results showed that NDRG4 mRNA expression was decreased in tumor specimens and significantly correlated with tumor differentiation, invasion and metastasis. Patients with tumor of reduced NDRG4 mRNA level had unfavorable disease-free and overall survival. Obesity was found to be adversely associated with disease-free and overall survival in tumors with reduced NDRG4 level, not in preserved NDRG4 level group, in both univariate and multivariate analysis. These data provided the first evidence that NDRG4 level in colorectal cancer could effectively stratify the prognostic value of obesity, which would better the understanding of the prognostic role of obesity in colorectal cancer. Our results also support the notion that the host-tumor interactions in colorectal cancer might influence tumor aggressiveness.

## INTRODUCTION

Colorectal cancer is one of the most common malignancies worldwide [1–3]. In recent years, the incidence rates of colorectal cancer are rapidly increasing in Asia [3–6]. In China, there has been a two- to four-fold increase in the incidence of colorectal cancer since the 1980s [7–9]. Accumulating epidemiologic evidence indicated that increased body mass index (BMI) was associated with an elevated risk of developing colorectal cancer [10–19]. And, the unfavorable increasing trend of colorectal cancer is thought to be, at least partly, due to the steep rise in prevalence of obesity [9, 20–22]. Therefore, better understanding the effect of obesity on colorectal cancer may lead to more effective cancer prevention strategy. However, till now, few investigations are available on the effect of BMI on the prognosis of colorectal cancer, with limited publications showed conflicting results [23–30]. Recently, a novel research paradigm in human malignancy found that the host-tumor interactions were able to modify tumor cell malignant behavior in humanmalignancies including colorectal cancer [31, 32]. In the link between excess energy balance and colorectal cancer malignant behavior, PI3K/AKT signaling pathway was found to be involved in by interacting with BMI [33–35]. Thus, the activation or inhibition of PI3K/AKT signaling pathway might determine the manner of colorectal cancer carcinogenesis in patients with different energy balance status.

N-Myc downstream-regulated gene (NDRG) family is comprised of four members, named NDRG1, NDRG2, NDRG3 and NDRG4 respectively, which share 57%–65% amino acid sequence homology [36, 37]. NDRG4, as the latest identified member, was previously considered to be specifically expressed in brain and heart tissue [36, 38–40]. However, we have demonstrated that NDRG4 protein expression was significantly decreased from normal mucosa, chronic colitis, ulcerative colitis, atypical hyperplasia to colorectal cancer tissues. In addition, NDRG4 in colorectal cancer was negatively correlated with PI3K/AKT activity and can significantly inhibit PI3K/AKT activity in tumor cell. These data suggested a tumor suppressive role of NDRG4 in carcinogenesis and progression, by the attenuation of PI3K/AKT signaling pathway [41]. Therefore, it is of theoretical rationality to deduce that the malignant behavior of colorectal cancer cells expressing NDRG4 might be from a PI3K/AKT-BMI interaction independent manner, whereas the malignant transformation of those with absent NDRG4 expression might be influenced by PI3K/AKT-BMI interaction. Considering the role of NDRG4 in the potential link with energy balance by PI3K/AKT attenuation, we hypothesized that activation of PI3K/AKT by absent NDRG4 expression might confer proliferative and progressive ability to colorectal cancer cells under excess energy balance status. Threrfore, it is possibile that the prognostic value of obesity in colorectal cancer might differ according to NDRG4 status.

To test this hypothesis, we investigated whether the associations of obesity with prognosis of colorectal cancer would be modified according to NDRG4 expression status in the present study.

## RESULTS

### Clinical and molecular characteristics of patients and specimens

In the study cohort consisted of 226 colorectal cancer patients, the mean age was 58.2 years, with a range of 21 to 81. According to our predetermined BMI categories (described in Materials and Methods), 52 (23.0%) were obese, 85 (37.6%) patients were overweight, 89 (39.4%) were normal weight. The 10-gene panel test found that 36 (16.0%) tumors were MSI-H while 190 (84.1%) were MSS. Mutated KRAS, BRAF and PIK3CA was found in 78 (34.5%), 37 (16.4%) and 33 (14.6%) tumors, while wild type of KRAS, BRAF and PIK3CA was found in 148 (65.5%), 189 (83.6%) and 193 (85.4%) tumors, respectively. Clinical characteristics were showed in Table 1.

**Table 1:** Statistical results of NDRG4 expression

Variable	*n*	NDRG4 mRNA expression		*P*
		Reduced (%)	Preserved (%)	
Total	226	160 (70.8%)	66 (29.2%)	
**Sex**				0.356*
Male	187	130 (69.5%)	57 (30.5%)	
Female	39	30 (76.9%)	9 (23.1%)	
**Age at diagnosis**				0.855*
≤ 60	128	90 (70.3%)	38 (29.7%)	
> 60	98	70 (71.4%)	28 (28.6%)	
**BMI**				
Normal weight	89	63 (70.8%)	26 (29.2%)	0.782*
Over weight	85	62 (72.9%)	23 (27.1%)	
Obese	52	35 (67.3%)	17 (32.7%)	
**Tumor location**				0.824*
Right	66	45 (68.2%)	21 (31.8%)	
Left	74	54 (73.0%)	20 (27.0%)	
Rectum	86	61 (70.9%)	25 (29.1%)	
**Tumor size**				0.783*
≤ 3.0 cm	42	29 (69.0%)	13 (31.0%)	
> 3.0 cm	184	131 (71.2%)	53 (28.8%)	
**Differentiation status**				< 0.001*
Well	46	24 (52.2%)	22 (47.8%)	
Moderately	99	67 (67.7%)	32 (32.3%)	
Poor	81	69 (85.2%)	12 (14.8%)	
**Depth of invasion**				< 0.001*
T_1_ + T_2_	77	32 (41.6%)	45 (58.4%)	
T_3_ + T_4_	149	128 (85.9%)	21 (14.1%)	
**Vascular invasion**				0.173^†^
Absent	216	151 (69.9%)	65 (30.1%)	
Present	10	9 (90.0%)	1 (10.0%)	
**Lymph node metastasis**				< 0.001*
Absent (N0)	97	52 (53.6%)	45 (46.4%)	
Present (N1–3)	129	108 (83.7%)	21 (16.3%)	
**Distant metastasis**				0.018^†^
Absent (M0)	202	138 (68.3%)	64 (31.7%)	
Present (M1)	24	22 (91.7%)	2 (8.3%)	
**TNM stage**				< 0.001^†^
I	56	28 (50.0%)	28 (50.0%)	
II	41	23 (56.1%)	18 (43.9%)	
III	105	87 (82.9%)	18 (17.1%)	
IV	24	22 (91.7%)	2 (8.3%)	
**MSI**				0.164*
MSS	190	138 (72.6%)	52 (27.4%)	
MSI-H	36	22 (61.1%)	14 (38.9%)	
**KRAS mutation**				0.811*
(−)	148	104 (70.3%)	44 (29.7%)	
(+)	78	56 (71.8%)	22 (28.2%)	
**BRAF mutation**				0.387*
(−)	189	136 (72.0%)	53 (28.0%)	
(+)	37	24 (64.9%)	13 (25.1%)	
**PIK3CA mutation**				0.881*
(−)	193	137 (71.0%)	56 (29.0%)	
(+)	33	23 (69.7%)	10 (30.3%)	

* *P* value when expression levels were compared using Pearson *χ*^2^ test

† *P* value when expression levels were compared using Fisher's exact test

### NDRG4 mRNA expression and its association with clinicopathologic characteristics of patients

As normalized to 18s rRNA, the RQ (standing for relative expression obtained by 2^−ΔΔCt^ method) of NDRG4 mRNA in colorectal cancer samples was 1.12 ± 0.15 (mean ± SD), while the relative NDRG4 mRNA expression detected in matched adjacent normal tissues was 1.87 ± 0.21. NDRG4 mRNA expression in colorectal cancer was significantly decreased compared with that in adjacent normal specimens and noncancerous control mucosa samples (*P* < 0.001). Based on the relative expression of NDRG4, we manually defined that the relative NDRG4 expression of 1.87 ± 0.21, which detected in adjacent normal tissues, as normal expression level of NDRG4 in colon mucosa, thus classified cancerous tissues into three groups: reduced expression of NDRG4 (less than 1.66), normal expression (1.66–2.08) and increased expression (over 2.08). For modeling purposes (because the number of tissues classified as increased expression of NDRG4 was small), cancerous tissues with normal and increased expression of NDRG4 were combined into a single group defined as having preserved NDRG4 expression. Therefore, 160 cases of colorectal cancer were defined as reduced NDRG4 expression group while 66 cases were defined as preserved expression group.

The correlation of NDRG4 mRNA levels with different clinicopathologic factors was shown in Table 1. NDRG4 mRNA expression was found to be associated with tumor cell differentiation, depth of wall invasion, vascular invasion, lymph node metastasis, distant metastases and TNM stage since reduced NDRG4 expression was more frequently to be detected in tumors with poor differentiation (*P* < 0.001), deep invasion (*P* < 0.001), lymph node metastasis (*P* < 0.001), distant metastases (*P* = 0.018) or advanced TNM stage (*P* < 0.001). While no statistically significant correlations were observed between NDRG4 mRNA expression and sex (*P* = 0.356), age at diagnosis (*P* = 0.855), BMI (*P* = 0.782), tumor location (*P* = 0.824), tumor size (*P* = 0.783), KRAS mutation (*P* = 0.811), BRAF mutation (*P* = 0.387), PIK3CA mutation (*P* = 0.881) or MSI (*P* = 0.164).

### NDRG4 stratifies the association of obesity with disease-free survival

**Figure 1:**
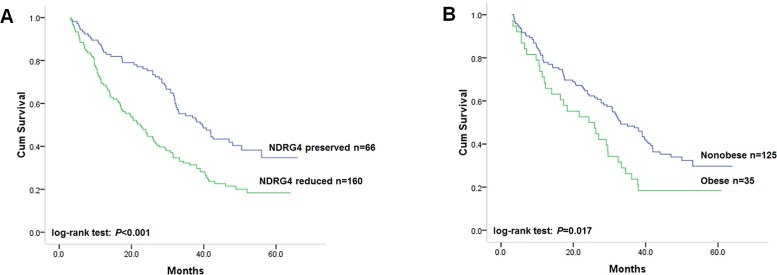
**(A)** Kaplan-Meier analysis on the association of NDRG4 mRNA expression with disease-free survival of all recruited patients; **(B)** Kaplan-Meier analysis on the association of obesity with disease-free survival in patients with tumor of reduced NDRG4 expression.

Kaplan-Meier analysis was used to evaluate the disease-free survival of patients with colorectal cancer and NDRG4 mRNA expression. Results showed that patients with preserved NDRG4 expression in colorectal cancer tissues had better disease-free survival in comparison to those with reduced NDRG4 expression (Figure 1A, log-rank test: *P* < 0.001), indicating that patients with colorectal cancer of reduced NDRG4 expression had a higher risk of tumor relapse compared with colorectal cancer of preserved NDRG4 expression. In addition, obesity (log-rank test: *P* = 0.032), tumor differentiation status (log-rank test: *P* < 0.001), lymph node metastasis (log-rank test: *P* < 0.001) and TNM stage (log-rank test: *P* < 0.001), MSI(log-rank test: *P* < 0.001), KRAS (log-rank test: *P* = 0.005), BRAF (log-rank test: *P* < 0.001) and PIK3CA(log-rank test: *P* < 0.001) mutations were also proved to be associated with disease-free survival of patients with colorectal cancer, which indicated that patients with obesity or patients with colorectal cancer of poor differentiation, advanced TNM stage, MSI, KRAS, BRAF or PIK3CA mutations had shorter disease-free survival and higher risk of relapse than those without. However, sex, age, tumor location, tumor size or vascular invasion had no prognostic value on disease-free survival of patients with colorectal cancer. Unadjusted hazard ratio (HR) was shown in Table 2. To verify the independent prognostic value of NDRG4 mRNA expression on disease-free survival of patients with colorectal cancer, cox proportional hazards model adjusted for sex, age, tumor location, tumor size, differentiation status, vascular invasion, TNM stage, KRAS, BRAF and PIK3CA mutations and MSI status was utilized to control for other prognostic factors. As a result, NDRG4 mRNA expression level was proved to be an independent prognostic factor after controlling for all other clinicopathologic factors. Adjusted HR was 1.00 (as a reference) in NDRG4 preserved expression patients, the adjusted HR of patients with colorectal cancer of reduced NDRG4 expression was 1.65 (95% CI: 1.18–2.30 *P* = 0.003, Table 2).

**Table 2:** Association of NDRG4 and clinical factors with disease-free survival of patients with CRC

	Unadjusted HR^*^(95% CI)	*P*	Adjusted HR^†^(95% CI)	*P*
NDRG4 expression	1.98 (1.41–2.76)	< 0.001	1.65 (1.18–2.30)	0.003
Sex	0.84 (0.57–1.26)	0.405	0.92 (0.64–1.45)	0.627
Age at diagnosis	1.12 (0.82–1.54)	0.468	1.05 (0.77–1.51)	0.793
Tumor location	1.25 (0.82–1.73)	0.763	1.03 (0.65–1.69)	0.902
Tumor size	1.53 (0.98–2.39)	0.061	1.26 (0.59–2.71)	0.511
Differentiation status	1.84 (1.17–2.90)	0.008	1.09 (0.65–1.94)	0.684
Vascular invasion	1.78 (0.91–3.51)	0.094	0.61 (0.29–1.34)	0.173
TNM stage	5.10 (2.79–9.33)	< 0.001	3.68 (1.53–8.92)	0.003
MSI	1.88 (1.36–2.59)	< 0.001	1.58 (1.11–2.24)	0.011
KRAS mutation	1.56 (1.14–2.14)	0.006	1.46 (1.04–2.04)	0.028
BRAF mutation	1.75 (1.28–2.39)	< 0.001	1.56 (1.12–2.16)	0.008
PIK3CA mutation	1.82 (1.31–2.52)	< 0.001	1.65 (1.17–2.33)	0.004

* Hazard ratios in univariate models

† Hazard ratios in multivariable models

Abbreviations: HR, hazard ratio; 95% CI, 95% confidence interval.

We have previously demonstrated that NDRG4 can significantly inhibit PI3K-AKT activity which has been considered to be involved in the pathogenetic link between excess energy balance and cancer [33]. Thus, considering the potential significant interaction between NDRG4 and obesity of patients with colorectal cancer, we examined the association of obesity with disease-free in strata of NDRG4 expression. Interestingly, we found that, among tumors with NDRG4 reduced expression, obesity was associated with significantly worse overall survival (Figure 1B, log-rank test: *P* = 0.017), with unadjusted HR to be 1.65 (95% CI: 1.09–2.51 *P* = 0.018). While obesity was not found to be significantly associated with disease-free in NDRG4 preserved group (Table 3). In multivariate analysis adjusted for sex, age, tumor location, tumor size, differentiation status, vascular invasion, TNM stage, KRAS, BRAF and PIK3CA mutations and MSI stutus, the association of disease-free survival with obesity was statistically significant for the adjusted HR was 1.71 (95% CI: 1.10–2.68 *P* = 0.018). These results indicated that obese patients with tumor of reduced NDRG4 expression, not preserved NDRG4 expression, had higher risk of tumor relapse compared with those nonobese patients.

**Table 3:** Association of obesity with disease-free survival according to NDRG4 level

	Unadjusted HR*(95% CI)	*P*	Adjusted HR^†^(95% CI)	*P*
Obese vs Nonobese (NDRG4 reduced)	1.65 (1.09–2.51)	0.018	1.71 (1.10–2.68)	0.018
Obese vs Nonobese (NDRG4 preserved)	1.47 (0.89–2.43)	0.131	1.45 (0.87–2.42)	0.156

* Hazard ratios in univariate models

† Hazard ratios in multivariable models

Abbreviations: HR, hazard ratio; 95% CI, 95% confidence interval.

### NDRG4 stratifies the association of obesity with overall survival

**Figure 2:**
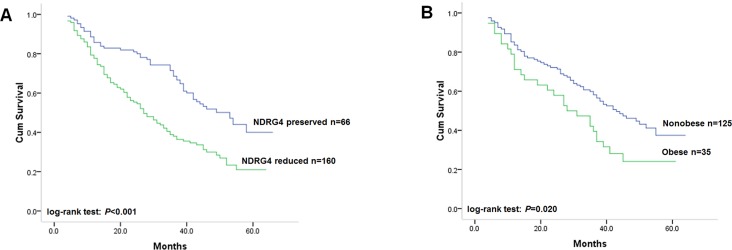
**(A)** Kaplan-Meier analysis on the association of NDRG4 mRNA expression with overall survival of all recruited patients; **(B)** Kaplan-Meier analysis on the association of obesity with overall survival in patients with tumor of reduced NDRG4 expression.

A statistically significant association between poor overall survival and reduced NDRG4 mRNA expression level was found in patients with colorectal cancer. Kaplan-Meier analysis for postoperative overall survival showed that patients with colorectal cancer of preserved NDRG4 expression had longer overall survival compared with patients with reduced expression of NDRG4 (Figure 2A, log-rank test: *P* < 0.001). Similar to results on disease-free survival, obesity (log-rank test: *P* = 0.036), tumor differentiation status (log-rank test: *P* < 0.001), lymph node metastasis (log-rank test: *P* < 0.001), TNM stage (log-rank test: *P* < 0.001), MSI(log-rank test: *P* < 0.001), KRAS(log-rank test: *P* = 0.014), BRAF(log-rank test: *P* = 0.003) and PIK3CA(log-rank test: *P* = 0.001) mutations were also proved to be prognostic factors for overall survival of patients with colorectal cancer. Patients with obesity and patients with colorectal cancer of poor differentiation, lymph node metastasis, advanced TNM stage, MSI, KRAS, BRAF or PIK3CA mutations had shorter overall survival. However, sex, age, tumor location, tumor size, vascular invasion or depth of invasion had no prognostic value on overall survival of patients with colorectal cancer. Unadjusted HR was shown in Table 4. Multivariate analysis showed that NDRG4 could be a prognostic factor for overall survival of patients with colorectal cancer independent of gender, age, differentiation status, TNM stage, KRAS, BRAF and PIK3CA mutations and MSI status. The adjusted HR of patients with colorectal cancer of reduced NDRG4 expression was 1.64 (95% CI: 1.13–2.36 *P* = 0.008, Table 4) with patients of preserved expression of NDRG4 to be reference. However, no statistically significant correlation between age, gender, tumor location, tumor size, vascular invasion or differentiation status and overall survival was found among patients with colorectal cancer.

**Table 4:** Association of NDRG4 and clinical factors with overall survival of patients with CRC

	Unadjusted HR* (95% CI)	*P*	Adjusted HR^†^(95% CI)	*P*
NDRG4 expression	2.05 (1.46–2.98)	< 0.001	1.64 (1.13–2.36)	0.008
Sex	0.73 (0.49–1.09)	0.128	0.83 (0.54–1.31)	0.391
Age at diagnosis	1.12 (0.81–1.57)	0.490	1.12 (0.79–1.58)	0.488
Tumor location	1.29 (0.86–1.96)	0.423	1.18 (0.69–2.04)	0.581
Tumor size	1.69 (0.83–3.72)	0.056	1.63 (0.75–3.58)	0.231
Differentiation status	2.33 (1.40–3.87)	0.001	1.25 (0.72–2.31)	0.453
Vascular invasion	1.91 (0.97–3.77)	0.061	0.67 (0.31–1.48)	0.265
TNM stage	5.76 (3.06–10.83)	< 0.001	3.91 (1.58–9.75)	0.003
MSI	1.90 (1.35–2.68)	< 0.001	1.57 (1.08–2.27)	0.018
KRAS mutation	1.51 (1.08–2.11)	0.016	1.44 (1.01–2.07)	0.045
BRAF mutation	1.65 (1.18–2.30)	0.003	1.49 (1.05–2.11)	0.026
PIK3CA mutation	1.83 (1.29–2.59)	0.001	1.64 (1.13–2.36)	0.008

* Hazard ratios in univariate models

† Hazard ratios in multivariable models

Abbreviations: HR, hazard ratio; 95% CI, 95% confidence interval.

As investigation on disease-free survival demonstrated that in only reduced NDRG4 group, not preserved NDRG4 group, obese patients with colorectal cancer had higher risk of tumor relapse compared with those nonobese patients. In order to further test our hypothesis that activation of PI3K/AKT by absent NDRG4 expression might confer proliferative and progressive ability to colorectal cancer cells under excess energy balance status, which would impact the prognostic value of obesity, we next evaluated the association of obesity with overall survival according to NDRG4 level. Univariate survival analysis showed that obese patients with colorectal cancer of reduced NDRG4 expression had unfavorable overall survival compared with nonobese patients (Figure 2B, log-rank test: *P* = 0.020). In contrast, those obese patients with tumors of preserved NDRG4 expression were not found to have significantly diverse survival pattern from nonobese patients (Table 5). In multivariate analysis, among patients with tumor of reduced NDRG4 expression, obesity was found to be independently associated with increased risk of death, with adjusted HR to be 1.67 (95% CI: 1.07–2.60, *P* = 0.023).

**Table 5:** Association of obesity with overall survival according to NDRG4 level

	Unadjusted HR* (95% CI)	*P*	Adjusted HR^†^(95% CI)	*P*
Obese vs Nonobese (NDRG4 reduced)	1.66 (1.06–2.58)	0.028	1.67 (1.07–2.60)	0.023
Obese vs Nonobese (NDRG4 preserved)	1.65 (0.96–2.84)	0.070	1.35 (0.75–2.42)	0.320

* Hazard ratios in univariate models

† Hazard ratios in multivariable models

Abbreviations: HR, hazard ratio; 95% CI, 95% confidence interval.

## DISCUSSION

The biological function of NDRG4 is largely unknown in colorectal cancer until now. We have recently demonstrated that NDRG4 protein expression was significantly decreased during carcinogenesis process of colorectal cancer. And the activity of PI3K/AKT signaling in colorectal cancer can be effectly attenuated by NDRG4 [41]. Considering the significant role of PI3K/AKT signaling in carcinogenesis and energy metabolism, we tested the hypothesis that NDRG4 expression levle in colorectal cancer might correlate to patient's energy metabolism and modify tumor cell malignant behavior in the present study. As a result, we found that reduced NDRG4 mRNA expression was associated with tumor progression, as well as unfavorable outcome independent of patients' clinical features and molecular variables including KRAS, BRAF and PIK3CA mutations and MSI status. These results were consistent with our previous invstigation on protein expression level of NDRG4 and further confirmed the tumor suppressor role of NDRG4 in colorectal cancer [41]. Although NDRG4 shares about 60% amino acid sequence homology with NDRG2, different from the widely tumor suppressive role of NDRG2, NDRG4 has been considered to be expressed mainly in brain and heart, and take part in the development of these organs. Until recently, investigations found that NDRG4 was overexpressed in human brain glioma and might promote tumor progression, indicating an oncogenetic role of NDRG4, which was opposed to the role of NDRG2 in human brain malignancy [42, 43]. However, we found that NDRG2 and NDRG4 mRNA expression was both decreased in colorectal cancer and adversely associated with clinical outcome within the same study cohort, indicating a tumor suppressive role of NDRG4 in colorectal cancer, which was similar to the role of NDRG2 in colorectal cancer [44]. These results suggested that NDRG4 and NDRG2 might both play tumor suppressive roles in colorectal cancer, which was different from their distinct roles in glioma. A recent comparative study on multitarget stool DNA test for colorectal cancer screening also showed that aberrant NDRG4 methylation was more likely to be detected in cancers, which further surport our results on the tumor suppressive role of NDRG4 in colorectal cancer [45].

Moreover, our investigation revealed that the prognostic impact of obesity on colorectal cancer relapse and overall mortility was significantly stratified by NDRG4 mRNA expression level. Specifically, among patients with tumor of reduce NDRG4 expression, obesity as associated with unfavorable disease-free and overall survival in both univariate and multivariate analysis. In contrast, no significant association of obesity with outcome was detected among patients with colorectal cancer of preserved NDRG4 expression. These results demonstrated that the adverse impact of obesity on colorectal cancer relapse and mortility was limited to patients with tumor of reduced NDRG4 expression, indicating that NDRG4 might correlate to patients' carcinogenesis and energy metabolism in determination of colorectal cancer clinical outcome.

Investigation on molecular alterations and clinical prognostic factors would provide potential effective solution for colorectal cancer management [44, 46–51]. In human malignancy, inactivation of tumor suppressor gene or activation of oncogene is thought to imply aggressive tumor behavior. However, human colorectal cancer has been found to develop through accumulation of multiple genetic alternations and epigenetic events. Therefore, in order to acquire malignant or aggressive features, tumors with preserved NDRG4 expression may have to acquire other aberrations which would confer even more malignant or aggressive biological behavior than those with reduced NDRG4 expression does. In the present study, we found a significant stratifying impact of NDRG4 on the association between obesity and clinical outcome in colorectal cancer. As NDRG4 has been demonstrated to significantly inhibit PI3K/AKT activity, energy balance may be correlated to malignant behavior of tumors with reduced NDRG4 expression. The modifying effect of NDRG4 of the prognostic value of obesity indicated that excess energy balance might be detrimental among patients with tumors of NDRG4 reduced expression, probably due to the activation of PI3K/AKT signaling pathway by absence of NDRG4. These results provided evidences for a possible interactive effect of NDRG4 in colorectal cancer and patient's energy balance status in determining tumor cell behavior. Therefore, our data support the hypothesis that tumor cells with reduced NDRG4 expression my depend on the interaction between PI3K/AKT activation and excess energy balance for malignant transformation and further progression, whereas those with preserved NDRG4 expression might undergo carcinogenesis and progression independent of this kind of host-tumor interaction. Although these data need to be confirmed by further investigation, our intriguing results would contribute to understanding the exact role of obesity in outcome of colorectal cancer and may have considerable clinical implications.

Our study has several strengths. It included a hospital-based prospective study cohort to explore the expression pattern of NDRG4 mRNA and its association with disease-free and overall survival. The hospital-based cohort facilitated us to acquire disease-free and overall survival information exactly. The sample size of the present study was large and homogeneous, with adequate follow-up time and intimate information on clinicopathological characteristics. To avoid mRNA expression being altered by preoperative neoadjuvant therapy, we limited the cohort to patients who were diagnosed before the year 2006, before neoadjuvant therapy were routinely used for colorectal cancer in our department. In addition, we also investigated critical molecular events such as KRAS, BRAF and PIK3CA mutations and MSI status, all of which have been associated with colorectal cancer prognosis in order to adjusted our results [52–54].

In summary, our large cohort propective study demonstrated that mRNA expression levels of NDRG4 in primary colorectal cancer might be a powerful, independent predictor of disease relapse and prognosis. Our results also provided the first evidence that NDRG4 had significant modifying effect on the prognostic value of obesity that obesity could be a significant independent predictor of unfavorable disease-free and overall survival among patients with colorectal cancer of reduced NDRG4 expression, but not among those with preserved NDRG4. These findings may have considerable clinical implications in obesity associated colorectal cancer management.

## MATERIALS AND METHODS

### Patients and specimens

This study was approved by the ethics committee of the Fourth Military Medical University. All patients involved provided full consent for the present study. The hospital-based study cohort including 226 patients consecutively diagnosed with colorectal cancer between January 2004 and December 2005 in Department of Gastrointestinal Surgery, Xijing Hospital, Fourth Military Medical University (Xi'an, China). Priori power calculation was not performed and the sample size was determined by access to a convenience sample of patients. Patients with following criteria were subsequently excluded: received treatment prior to surgery including neoadjuvant chemotherapy; harvested insufficient specimens for RNA isolation; diagnosed as gastrointestinal stromal tumor or lymphoma; diagnosed with additional cancers; refused consent. Clinicopathologic information and follow-up data of the remaining 226 patients were prospectively entered into a database, which was under a close follow-up scheme and updated with respect to survival status every three month by telephone visit and questionnaire letters. Thirty-six noncancerous healthy colon mucosa tissues obtained from patients underwent surgery or endoscopy without malignancy served as control. All the fresh tissues were obtained within 10 minutes after surgical removal and put into liquid nitrogen for 10 min, then into a −80°C ultra-freezer for mRNA isolation. All the specimens had been histologically diagnosed by Department of Pathology, Xijing Hospital, Fourth Military Medical University. Study physicians who reviewed all the records of colorectal cancer and recorded data into database were totally blind to exposure data. Clinicopathologic information of all the 226 patients was available.

### Measurement of endpoints

In the present study, disease-free survival is defined as the time elapsed from surgery to the first occurrence of any of the following events: colorectal cancer distant metastasis; recurrence of colorectal cancer; development of second non-colorectal malignancy excluding basal cell carcinomas of the skin and carcinoma *in situ* of the cervix; or death from any cause. The diagnosis of disease relapse was based on the imaging method such as ultrasonography, computed tomography, magnetic resonance imaging and position emission tomography, if possible, cytologic analysis or biopsy. Overall survival is defined as the time elapsed from surgery to death of patients with colorectal cancer. Death of participants was ascertained by reporting from the family and verified by review of public records. The disease-free and overall survival status was assigned by physicians blinded to other clinicopathologic and NDRG4 mRNA expression information.

### Assessment of body mass index and smoking status

To insure the consistency of body mass index (BMI), weight (kilograms) and height (meters) of patients were measured and recorded at uniform time points relative to diagnosis and surgery by trained staff. These measurements were then transferred to trained personnel to calculate BMI by taking the body weight in kilograms divided by height in meters squared. For the present study, participants with BMI less than 18.5 kg/m^2^ had been defined as underweight and excluded, other participants were then categorized according to the World Health Organization (WHO) classification for Asian populations, normal weight (18.5 kg/m^2^ ≤ BMI < 23.0 kg/m^2^), overweight (23.0 kg/m^2^ ≤ BMI < 27.5 kg/m^2^) and obese (BMI ≥ 27.5 kg/m^2^).

### RNA extraction and real-time polymerase chain reaction

When patients recruitment accomplished, total RNA from all the 226 colorectal cancer tissue and matched adjacent normal tissue specimens together with 36 noncancerous healthy colon mucosa tissues was purified as recommended by the manufacturer using Trizol reagent (Invitrogen, Carlsbad, CA). cDNA synthesis was performed using approximately 5 μg RNA per 20 μL using a cDNA reverse transcription kit (Fermentas). Real-time PCR was performed on an ABI 7500 system (Applied Biosystems) using SYBR Green I (TAKARA). Primers were designed using Primer Express v3.0 Software. NDRG4 primers were: forward 5′-GGAGGTTGTCTCTTTGGTCAAGGT-3′, reverse 5′-CTCATGACAGCAGCCACCAGAAT -3′. The internal control 18S rRNA primers were: forward 5′- CGCCGCTAGAGGTGAAATTC -3′ and reverse 5′- TTGGCAAATGCTTTCGCTC -3′. After first strand synthesis, an equivalent of 50 ng of starting total cellular RNA (1/10 of the cDNA reaction) was added to two duplicate PCR reactions containing 12.5 μL SybrGreen mix, 0.5 μL SybrGreen rox, 100 nmol/L forward primer, and 100 nmol/L reverse primer in a final volume of 25 μL. Each sample was used in a single reaction that cycled at 95°C for 10 min (to activate enzyme), followed by 45 cycles of 95°C for 10 s and 60°C for 34 s on an ABI SDS 7500 system (Applied Biosystems). The mRNA expression of NDRG4 was analyzed using the 2^−ΔΔCt^ method. Fluorescent data were converted into RQ measurements, which stand for relative expression automatically by the SDS system software and exported to Microsoft Excel. NDRG4 mRNA levels were normalized to 18S rRNA. Thermal dissociation plots were examined for biphasic melting curves, indicative of whether primer-dimers or other nonspecific products could be contributing to the amplification signal. Sequencing of randomly selected real-time PCR product was utilized to insure the quality of real-time PCR.

### DNA extraction, microsatellite instability (MSI), pyrosequencing of KRAS, BRAF and PIK3CA analysis

MSI status was determined via testing on a 10-gene panel in tumor DNA using 10 microsatellite markers (BAT25, BAT26, BAT40, MYCL, D5S346, D17S250, ACTC, D18S55, D10S197, and BAT34C4) as described in previous study [56]. In brief, tumors with MSI-high/ microsatellite stability (MSI-H) was defined if instability was observed for ≥ 30% of markers, while and MSI-low/microsatellite stability (MSS) was defined if instability was observed for < 30% of the markers. And we also performed PCR and pyrosequencing targeted for KRAS (codons 12 and 13), BRAF (codon 600) and PIK3CA (exons 9 and 20) [57–59].

### Statistical analysis

Statistical analysis was carried out by the statistical package SPSS (version l3.0). Associations between NDRG4 mRNA expression and categorical variables were analyzed by Pearson *χ*^2^ test or Fisher's exact test, as appropriate. Survival curves were estimated using the Kaplan-Meier method, and differences in survival distributions were evaluated by the log-rank test. Cox's proportional hazards modeling of factors potentially related to survival was performed in order to identify which factors might have a significantly independent influence on survival. Differences with a *P* value of 0.05 or less were considered to be statistically significant.

## References

[1] Siegel R, Naishadham D, Jemal A (2013). Cancer statistics, 2013. CA: a cancer journal for clinicians.

[2] Center MM, Jemal A, Smith RA, Ward E (2009). Worldwide variations in colorectal cancer. CA: a cancer journal for clinicians.

[3] Jemal A, Bray F, Center MM, Ferlay J, Ward E, Forman D (2011). Global cancer statistics. CA: a cancer journal for clinicians.

[4] Matsuo K, Mizoue T, Tanaka K, Tsuji I, Sugawara Y, Sasazuki S, Nagata C, Tamakoshi A, Wakai K, Inoue M, Tsugane S (2012). Development and Evaluation of Cancer Prevention Strategies in J. Association between body mass index and the colorectal cancer risk in Japan: pooled analysis of population-based cohort studies in Japan. Annals of oncology.

[5] Sung JJ, Lau JY, Goh KL, Leung WK, Asia Pacific Working Group on Colorectal C (2005). Increasing incidence of colorectal cancer in Asia: implications for screening. The lancet oncology.

[6] Sung JJ, Lau JY, Young GP, Sano Y, Chiu HM, Byeon JS, Yeoh KG, Goh KL, Sollano J, Rerknimitr R, Matsuda T, Wu KC, Ng S, Leung SY, Makharia G, Chong VH (2008). Asia Pacific consensus recommendations for colorectal cancer screening. Gut.

[7] Deng SX, Gao J, An W, Yin J, Cai QC, Yang H, Li ZS (2011). Colorectal cancer screening behavior and willingness: an outpatient survey in China. World journal of gastroenterology.

[8] Zhang S, Cui Y, Weng Z, Gong X, Chen M, Zhong B (2009). Changes on the disease pattern of primary colorectal cancers in Southern China: a retrospective study of 20 years. International journal of colorectal disease.

[9] Li M, Gu J (2005). Changing patterns of colorectal cancer in China over a period of 20 years. World journal of gastroenterology.

[10] Odegaard AO, Koh WP, Yu MC, Yuan JM (2011). Body mass index and risk of colorectal cancer in Chinese Singaporeans: the Singapore Chinese Health Study. Cancer.

[11] Renehan AG, Soerjomataram I, Tyson M, Egger M, Zwahlen M, Coebergh JW, Buchan I (2010). Incident cancer burden attributable to excess body mass index in 30 European countries. International journal of cancer.

[12] Oxentenko AS, Bardia A, Vierkant RA, Wang AH, Anderson KE, Campbell PT, Sellers TA, Folsom AR, Cerhan JR, Limburg PJ (2010). Body size and incident colorectal cancer: a prospective study of older women. Cancer prevention research.

[13] Huxley RR, Ansary-Moghaddam A, Clifton P, Czernichow S, Parr CL, Woodward M (2009). The impact of dietary and lifestyle risk factors on risk of colorectal cancer: a quantitative overview of the epidemiological evidence. International journal of cancer.

[14] Hughes LA, Simons CC, van den Brandt PA, Goldbohm RA, van Engeland M, Weijenberg MP (2011). Body size and colorectal cancer risk after 16. 3 years of follow-up: an analysis from the Netherlands Cohort Study. American journal of epidemiology.

[15] Russo A, Franceschi S, La Vecchia C, Dal Maso L, Montella M, Conti E, Giacosa A, Falcini F, Negri E (1998). Body size and colorectal-cancer risk. International journal of cancer.

[16] Adams KF, Leitzmann MF, Albanes D, Kipnis V, Mouw T, Hollenbeck A, Schatzkin A (2007). Body mass and colorectal cancer risk in the NIH-AARP cohort. American journal of epidemiology.

[17] Campbell PT, Cotterchio M, Dicks E, Parfrey P, Gallinger S, McLaughlin JR (2007). Excess body weight and colorectal cancer risk in Canada: associations in subgroups of clinically defined familial risk of cancer. Cancer epidemiology, biomarkers & prevention.

[18] Giacosa A, Franceschi S, La Vecchia C, Favero A, Andreatta R (1999). Energy intake, overweight, physical exercise and colorectal cancer risk. European journal of cancer prevention.

[19] Nock NL, Thompson CL, Tucker TC, Berger NA, Li L (2008). Associations between obesity and changes in adult BMI over time and colon cancer risk. Obesity.

[20] Meng W, Cai SR, Zhou L, Dong Q, Zheng S, Zhang SZ (2009). Performance value of high risk factors in colorectal cancer screening in China. World journal of gastroenterology.

[21] Hu FB, Liu Y, Willett WC (2011). Preventing chronic diseases by promoting healthy diet and lifestyle: public policy implications for China. Obesity reviews.

[22] Xi B, Liang Y, He T, Reilly KH, Hu Y, Wang Q, Yan Y, Mi J (2012). Secular trends in the prevalence of general and abdominal obesity among Chinese adults, 1993–2009. Obesity reviews.

[23] Baade PD, Meng X, Youl PH, Aitken JF, Dunn J, Chambers SK (2011). The impact of body mass index and physical activity on mortality among patients with colorectal cancer in Queensland, Australia. Cancer epidemiology, biomarkers & prevention.

[24] Meyerhardt JA, Niedzwiecki D, Hollis D, Saltz LB, Mayer RJ, Nelson H, Whittom R, Hantel A, Thomas J, Fuchs CS, Cancer, Leukemia Group B. (2008). Impact of body mass index and weight change after treatment on cancer recurrence and survival in patients with stage III colon cancer: findings from Cancer and Leukemia Group B 89803. Journal of clinical oncology.

[25] Dignam JJ, Polite BN, Yothers G, Raich P, Colangelo L, O'Connell MJ, Wolmark N (2006). Body mass index and outcomes in patients who receive adjuvant chemotherapy for colon cancer. Journal of the National Cancer Institute.

[26] Prizment AE, Flood A, Anderson KE, Folsom AR (2010). Survival of women with colon cancer in relation to precancer anthropometric characteristics: the Iowa Women's Health Study. Cancer epidemiology, biomarkers & prevention.

[27] Calle EE, Rodriguez C, Walker-Thurmond K, Thun MJ (2003). Overweight, obesity, and mortality from cancer in a prospectively studied cohort of U.S. adults. The New England journal of medicine.

[28] Murphy TK, Calle EE, Rodriguez C, Kahn HS, Thun MJ (2000). Body mass index and colon cancer mortality in a large prospective study. American journal of epidemiology.

[29] Sinicrope FA, Foster NR, Sargent DJ, O'Connell MJ, Rankin C (2010). Obesity is an independent prognostic variable in colon cancer survivors. Clinical cancer research.

[30] Schmitz KH, Neuhouser ML, Agurs-Collins T, Zanetti KA, Cadmus-Bertram L, Dean LT, Drake BF (2013). Impact of obesity on cancer survivorship and the potential relevance of race and ethnicity. Journal of the National Cancer Institute.

[31] Morikawa T, Kuchiba A, Yamauchi M, Meyerhardt JA, Shima K, Nosho K, Chan AT, Giovannucci E, Fuchs CS, Ogino S (2011). Association of CTNNB1 (beta-catenin) alterations, body mass index, and physical activity with survival in patients with colorectal cancer. JAMA.

[32] Kuchiba A, Morikawa T, Yamauchi M, Imamura Y, Liao X, Chan AT, Meyerhardt JA, Giovannucci E, Fuchs CS, Ogino S (2012). Body mass index and risk of colorectal cancer according to fatty acid synthase expression in the nurses' health study. Journal of the National Cancer Institute.

[33] Engelman JA, Luo J, Cantley LC (2006). The evolution of phosphatidylinositol 3-kinases as regulators of growth and metabolism. Nature reviews Genetics.

[34] Greenhill C (2015). Obesity: Inhibiting PI3K reduces body weight in obese mice. Nat Rev Endocrinol.

[35] Ortega-Molina A, Lopez-Guadamillas E, Mattison JA, Mitchell SJ, Munoz-Martin M, Iglesias G, Gutierrez VM, Vaughan KL, Szarowicz MD, Gonzalez-Garcia I, Lopez M, Cebrian D, Martinez S, Pastor J, de Cabo R, Serrano M (2015). Pharmacological Inhibition of PI3K Reduces Adiposity and Metabolic Syndrome in Obese Mice and Rhesus Monkeys. Cell metabolism.

[36] Zhou RH, Kokame K, Tsukamoto Y, Yutani C, Kato H, Miyata T (2001). Characterization of the human NDRG gene family: a newly identified member, NDRG4, is specifically expressed in brain and heart. Genomics.

[37] Qu X, Zhai Y, Wei H, Zhang C, Xing G, Yu Y, He F (2002). Characterization and expression of three novel differentiation-related genes belong to the human NDRG gene family. Molecular and cellular biochemistry.

[38] Ding W, Zhang J, Yoon JG, Shi D, Foltz G, Lin B (2012). NDRG4 is downregulated in glioblastoma and inhibits cell proliferation. Omics.

[39] Okuda T, Kokame K, Miyata T (2008). Differential expression patterns of NDRG family proteins in the central nervous system. The journal of histochemistry and cytochemistry.

[40] Ohki T, Hongo S, Nakada N, Maeda A, Takeda M (2002). Inhibition of neurite outgrowth by reduced level of NDRG4 protein in antisense transfected PC12 cells. Brain research Developmental brain research.

[41] Chu D, Zhang Z, Zhou Y, Li Y, Zhu S, Zhang J, Zhao Q, Ji G, Wang W, Zheng J (2015). NDRG4, a novel candidate tumor suppressor, is a predictor of overall survival of colorectal cancer patients. Oncotarget.

[42] Schilling SH, Hjelmeland AB, Radiloff DR, Liu IM, Wakeman TP, Fielhauer JR, Foster EH, Lathia JD, Rich JN, Wang XF, Datto MB (2009). NDRG4 is required for cell cycle progression and survival in glioblastoma cells. The Journal of biological chemistry.

[43] Li W, Chu D, Chu X, Meng F, Wei D, Li H, Sun B (2011). Decreased expression of NDRG2 is related to poor overall survival in patients with glioma. Journal of clinical neuroscience.

[44] Chu D, Zhang Z, Li Y, Wu L, Zhang J, Wang W, Zhang J (2011). Prediction of colorectal cancer relapse and prognosis by tissue mRNA levels of NDRG2. Molecular cancer therapeutics.

[45] Imperiale TF, Ransohoff DF, Itzkowitz SH, Levin TR, Lavin P, Lidgard GP, Ahlquist DA, Berger BM (2014). Multitarget stool DNA testing for colorectal-cancer screening. The New England journal of medicine.

[46] Chu D, Zheng J, Li J, Li Y, Zhang J, Zhao Q, Wang W, Ji G (2014). MicroRNA-630 is a prognostic marker for patients with colorectal cancer. Tumour biology.

[47] Chu D, Zhao Z, Zhou Y, Li Y, Li J, Zheng J, Zhao Q, Wang W (2012). Matrix metalloproteinase-9 is associated with relapse and prognosis of patients with colorectal cancer. Annals of surgical oncology.

[48] Chu D, Zhang Z, Zhou Y, Wang W, Li Y, Zhang H, Dong G, Zhao Q, Ji G (2011). Notch1 and Notch2 have opposite prognostic effects on patients with colorectal cancer. Annals of oncology.

[49] Chu D, Zhang Z, Li Y, Zheng J, Dong G, Wang W, Ji G (2011). Matrix metalloproteinase-9 is associated with disease-free survival and overall survival in patients with gastric cancer. International journal of cancer.

[50] Chu D, Li Y, Wang W, Zhao Q, Li J, Lu Y, Li M, Dong G, Zhang H, Xie H, Ji G (2010). High level of Notch1 protein is associated with poor overall survival in colorectal cancer. Annals of surgical oncology.

[51] Chu D, Zhou Y, Zhang Z, Li Y, Li J, Zheng J, Zhang H, Zhao Q, Wang W, Wang R, Ji G (2011). Notch1 expression, which is related to p65 Status, is an independent predictor of prognosis in colorectal cancer. Clinical cancer research.

[52] Walter V, Jansen L, Hoffmeister M, Brenner H (2014). Smoking and survival of colorectal cancer patients: systematic review and meta-analysis. Ann Oncol.

[53] Takahashi N, Yamada Y, Furuta K, Honma Y, Iwasa S, Takashima A, Kato K, Hamaguchi T, Shimada Y (2014). Serum levels of hepatocyte growth factor and epiregulin are associated with the prognosis on anti-EGFR antibody treatment in KRAS wild-type metastatic colorectal cancer. British journal of cancer.

[54] Alonso-Espinaco V, Cuatrecasas M, Alonso V, Escudero P, Marmol M, Horndler C, Ortego J, Gallego R, Codony-Servat J, Garcia-Albeniz X, Jares P, Castells A, Lozano JJ, Rosell R, Maurel J (2014). RAC1b overexpression correlates with poor prognosis in KRAS/BRAF WT metastatic colorectal cancer patients treated with first-line FOLFOX/XELOX chemotherapy. European journal of cancer.

[55] Therasse P, Arbuck SG, Eisenhauer EA, Wanders J, Kaplan RS, Rubinstein L, Verweij J, Van Glabbeke M, van Oosterom AT, Christian MC, Gwyther SG (2000). New guidelines to evaluate the response to treatment in solid tumors. European Organization for Research and Treatment of Cancer, National Cancer Institute of the United States, National Cancer Institute of Canada. Journal of the National Cancer Institute.

[56] Newcomb PA, Baron J, Cotterchio M, Gallinger S, Grove J, Haile R, Hall D, Hopper JL, Jass J, Le Marchand L, Limburg P, Lindor N, Potter JD, Templeton AS, Thibodeau S, Seminara D (2007). Colon Cancer Family Registry: an international resource for studies of the genetic epidemiology of colon cancer. Cancer epidemiology, biomarkers & prevention.

[57] Ogino S, Kawasaki T, Brahmandam M, Yan L, Cantor M, Namgyal C, Mino-Kenudson M, Lauwers GY, Loda M, Fuchs CS (2005). Sensitive sequencing method for KRAS mutation detection by Pyrosequencing. The Journal of molecular diagnostics.

[58] Ogino S, Kawasaki T, Kirkner GJ, Loda M, Fuchs CS (2006). CpG island methylator phenotype-low (CIMP-low) in colorectal cancer: possible associations with male sex and KRAS mutations. The Journal of molecular diagnostics.

[59] Nosho K, Kawasaki T, Ohnishi M, Suemoto Y, Kirkner GJ, Zepf D, Yan L, Longtine JA, Fuchs CS, Ogino S (2008). PIK3CA mutation in colorectal cancer: relationship with genetic and epigenetic alterations. Neoplasia.

